# A Call for the Aggressive Treatment of Oligometastatic and Oligo-Recurrent Non-Small Cell Lung Cancer

**DOI:** 10.1155/2012/480961

**Published:** 2012-10-17

**Authors:** Pretesh R. Patel, David S. Yoo, Yuzuru Niibe, James J. Urbanic, Joseph K. Salama

**Affiliations:** ^1^Department of Radiation Oncology, Duke University, P.O. Box 3085, Durham NC 27713, USA; ^2^Department of Radiology and Radiation Oncology, Kitasato University School of Medicine, Sagamihara 252-0374, Japan; ^3^Department of Radiation Oncology, Wake Forest University, Winston-Salem, NC 27157, USA

## Abstract

Metastatic non-small cell lung cancer (NSCLC) carries a dismal prognosis. Clinical evidence suggests the existence of an intermediate, or oligometastatic, state when metastases are limited in number and/or location. In addition, following initial curative therapy, many patients present with limited metastatic disease, or oligo-recurrence. Metastasis-directed, anti-cancer therapies may benefit these patients. A growing evidence-base supports the use of hypofractionated, image-guided radiotherapy (HIGRT) for a variety of malignant conditions including inoperable stage I NSCLC and many metastatic sites. When surgical resection is not possible, HIGRT offers an effective alternative for local treatment of limited metastatic disease. Early studies have produced promising results when HIGRT was delivered to all known sites of disease in patients with oligometastatic/oligo-recurrent NSCLC. In a population of patients formerly considered rapidly terminal, these studies report five year overall survival rates of 13–22%. HIGRT for metastatic NSCLC warrants further study. We call for large, intergroup, and even international randomized trials incorporating HIGRT and other metastasis-directed therapies into the treatment of patients with oligometastatic/oligo-recurrent NSCLC.

## 1. Introduction

Lung cancer is the most lethal malignant tumor. Affecting over one million people each year, it results in approximately 951,000 deaths [1]. Eighty-five percent of lung cancer patients have non-small cell lung cancer (NSCLC), and about 40% of those will present with distant metastatic disease [2]. The current standard therapy for most metastatic NSCLC patients is doublet chemotherapy. Contemporary regimens, such as cisplatin and docetaxel, demonstrate superior outcomes compared to regimens of the last decade [3, 4]. Even with the most effective cytotoxic agents only 30% of patients respond to therapy and the median survival from diagnosis is approximately 1 year [5–7]. Worse still, the response to second line therapy is poor (7–11%) with a median survival of 8 months at best [8, 9]. Herein, we call for the systematic study of new approaches and integration of all available therapeutic modalities in the management of this humbling disease.

## 2. Oligometastases and Oligo-Recurrence

It has been proposed that the natural history of metastatic spread may proceed stepwise, and there exists an oligometastatic state when metastases are limited in number and/or location and therefore amenable to loco-regional therapy [10]. In other cases, when subclinical disease is eradicated by systemic therapy, the clinically apparent metastases may be considered “residual” oligometastases, which may serve as a nidus for further dissemination [11]. Furthermore, following initial curative therapy, a large number of patients will recur, and many will have recurrences limited in number and destination organ, that is, oligo-recurrence [12]. The key distinction between oligo-recurrence and oligometastasis is that the primary tumor is controlled in the former and a small institutional series suggests more favorable prognosis [13]. Metastasis-directed anti-cancer therapies may benefit patients with *de novo* oligometastases, *induced* oligometastases, or oligo-recurrence.

A fact not often appreciated is that the oligometastatic/oligo-recurrent phenotype is common. Widespread use of more sensitive staging studies, such as PET/CT, has led to a growing incidence of stage IV NSCLC [14]. In addition, patients receiving systemic therapy for stage IV NSCLC often progress only in sites of known metastases. An analysis of metastatic NSCLC patients treated in a phase II protocol with oxaliplatin and paclitaxel at the University of Chicago, found that 50% (19/38) of patients had stable or progressive disease *only in sites that were initially involved with tumor without developing new metastatic lesions *[15]. This number grew to 65% (11/17) in the subset with 4 or fewer metastases. Similarly, an analysis of patients with limited metastatic NSCLC from the University of Colorado demonstrated that the patterns of progression are primarily within known sites of disease [16]. 

Tailored systemic therapy and targeted agents may further improve the control of subclinical disease and induce an oligometastatic state. Non-squamous NSCLC responds favorably to pemetrexed-based systemic regimens [17, 18]. The addition of a targeted antiangiogenic agent, bevacizumab, to carboplatin and paclitaxel [19] has resulted in improved survival. Patients with epidermal growth factor receptor (EGFR) mutations have superior progressive free survival when treated with EGFR inhibitors [20]. Similarly, identification of the EML4-ALK mutation results in superior survival when crizotinib, a small molecule ALK inhibitor, is included in the systemic therapy regimen [21]. Whole genome sequencing of NSCLC is underway [22] and may lead to identification of novel subtypes and personalized therapies.

Selected patients with limited metastatic disease have achieved cure and prolonged palliation with local and regional treatment. For example, resection of brain [33], lung [34], liver [35], and adrenal [36] metastases have resulted in long term cure of patients with metastatic NSCLC. In addition, aggressive treatment of intracranial metastases with stereotactic radiosurgery (SRS) has resulted in high long term disease control rates [37]. These long term survivors are clinical proof of the oligometastatic/oligo-recurrent state. Moreover, with improving systemic therapies, the control of oligometastases will play a larger role in determining patient outcome. Even if cure rates remain low, local treatment could prevent or ameliorate morbidity related to local tumor proliferation.

## 3. Hypofractionated, Image-Guided Radiotherapy (HIGRT)

For patients who are not candidates for surgical excision of metastatic disease, radiotherapy (RT) is an effective alternative local therapy. Fractionated RT has long played a role in the palliation of metastatic NSCLC. Technological improvements over the past decade have led to modern RT delivery systems capable of unprecedented precision and accuracy. Stereotactic, high-dose, single fraction brain irradiation, or stereotactic radiosurgery (SRS), was once considered the vanguard of RT. Today, improvements in tumor target delineation, RT dosimetry, respiratory motion management, and tumor targeting have led to the proliferation of brain SRS and stereotactic body radiotherapy (SBRT). SBRT, or stereotactic ablative radiotherapy (SABR), is perhaps more aptly described as hypofractionated, image-guided radiotherapy (HIGRT) now that stereotactic frames are rarely used.

An exploding body of literature supports the use of HIGRT for a variety of malignant conditions. Brain SRS is associated with excellent local control without significant toxicity. In fact, 80–95% of tumors less than 2 cm were permanently controlled by single doses of 18–20 Gy regardless of tumor type [38]. The local control for lung lesions is similarly excellent. Phase I and II American studies have demonstrated greater than 90% primary tumor control following HIGRT for medically inoperable NSCLC [39]. Japanese studies have also showed overall local control of 89.6% with an NCI-CTC grade 3–5 complication rate of only 2.1% [40]. Evidence-based guidelines now recommend definitive lung HIGRT for medically inoperable stage I NSCLC [41]. Promising results are also emerging to support the use of definitive HIGRT for prostate cancer [42, 43], and inoperable pancreatic cancer [44]. Lung [45, 46], liver [47, 48], and spinal [49–53] metastases have been effectively treated with HIGRT, including classically radioresistant histologies such as melanoma [54] and renal cell carcinoma [55]. What is striking in all these studies is that a high probability of durable treated metastasis control is possible with the use of high conformal, precisely targeted, (usually) substantially hypofractionated treatment courses regardless of metastatic site or histology. Equally encouraging has been the relative limited toxicity reported with these treatments. 

Biologically, it is not clear why hypofractionated radiotherapy results in high tumor control rates. Hypofractionated radiotherapy has radiobiological advantages over standard fractionated RT including a greater potential cell kill and reduction in the deleterious effect of tumor proliferation during RT. Large radiation doses are thought to not only enhance tumor cell kill, but also engage sphingomyelin-based endothelial mechanisms of tumor control [56, 57]. Additionally, recent reports have identified immune-mediated mechanisms that may play a key role in controlling tumors following hypofractionated RT [58, 59]. The use of ablative radiotherapy in concert with immunomodulatory therapies have demonstrated an abscopal effect, that is, a response in nonirradiated metastases [60, 61]. This abscopal effect may become particularly relevant in the treatment of oligometastatic disease where the potentiation of an immune response could be particularly efficacious.

## 4. Metastasis-Directed HIGRT

Data are now beginning to emerge that the aggressive treatment of both the primary tumor and metastases with RT as an integral component can result in improved outcomes. Median survival following conventional radiotherapy for brain metastases is 3–6 months and 1-year survival of 8% in a large retrospective series [62]. Even those with only 1-2 brain metastases have a 2-year survival of only 6% [63]. A number of studies have focused on the subgroup of patients with limited intracranial metastases from NSCLC (Table 1). In these studies, aggressive treatment of metachronous brain metastases in NSCLC patients, that is oligo-recurrence, without extracranial disease produced 5-year survival of 13.2% [23]. Additionally, in those with synchronous solitary brain metastases, aggressive treatment of intracranial metastases with radiosurgery as well aggressive treatment of intrathoracic disease resulted in 21% 5-year survival [24]. A recent review on this topic concludes that aggressive brain and thoracic treatment should be offered to these patients [64].

Reports, primarily from single institutions, have demonstrated favorable outcomes when patients with limited extracranial metastatic NSCLC received aggressive therapy to all known cancer sites (Table 1) [13, 26–29]. An analysis from the University of Rochester reported median survivals of patients with limited metastatic NSCLC treated with HIGRT to be similar to that of stage III NSCLC patients and exhibiting 5-year survival of 14% [26]. Twenty-five patients from the University of Chicago with a median of two extracranial metastases underwent HIGRT and had median survival similar to that seen in stage III patients at 23-month and 18-month overall survival of 53% [28]. Interestingly, those treated with prior systemic therapy, those progressing through chemotherapy immediately prior to HIGRT, and nonadenocarcinoma histology were associated with worse outcomes. 

Looking at all these data, one message stands clear; all known cancer sites must be treated to benefit patients with limited metastatic NSCLC. An analysis of the M.D. Anderson Cancer Center Registry, presented at ASCO 2008 (abstr no. 19020), supports aggressive treatment of primary tumors and regional nodes in patients with metastatic NSCLC. This study found that patients with solitary brain metastases from NSCLC who received curative-intent thoracic locoregional treatment with either surgery or concomitant chemoradiotherapy median survival improved from 7 to 30 months (*P* = 0.00186), compared to those who did not. Additionally, this survival advantage was not statistically significant in patients with untreated extracranial metastases. Furthermore, patients with solitary brain metastases treated with surgical resection [13]or radiosurgery [24]significantly benefited from treatment to the primary tumor in addition to aggressive treatment of metastatic disease. This highlights the need to treat all known metastatic deposits whenever possible. 

## 5. Metastasis-Directed HIGRT: Prospective Trials 

Based on the promising data, it is clear that further study is needed to carefully integrate these novel RT techniques with standard systemic therapy platforms for patients with metastatic NSCLC. Attempts have been made to prospectively study HIGRT (Table 2). Each study has asked different questions so it is worth reviewing each in some detail.

The NCCTG initiated a randomized phase III study to test the hypothesis that RT to all known sites of disease following 4–6 cycles of systemic therapy in NSCLC patients with one to three metastatic sites would result in improved overall survival [30]. Following the completion of non-standardized systemic therapy, patients were randomized to observation or RT to all known sites of disease. The RT schema was 60 Gy in 2 Gy fractions or 45 Gy in 3 Gy fractions. The study was closed due to poor accrual. This was likely due to randomization following all chemotherapy, a time when patients and physicians are looking forward to an end of treatment. Additionally, the protracted courses of radiation over a six-week time span with historically limited control rates may have contributed. 

 The University of Chicago initiated a randomized phase II study in patients with 1–5 NSCLC metastases, testing the hypothesis that HIGRT to all known sites of metastatic disease during the third and fourth cycles of systemic therapy would improve progression-free and overall survival [31]. Based on prior institutional studies, cisplatin and docetaxel were used as the chemotherapy backbone and RT was given in 5 Gy fractions to a total dose of 50 Gy. Additionally, conventionally fractionated radiotherapy (60 Gy in 2 Gy fractions) was allowed when combined with systemic therapy for stage III-type intra-thoracic disease. Different from the NCCTG study, this study randomized patients prior to any therapy. This study too, had difficulty accruing, and closed prior to meeting the accrual goal. 

Currently, a single arm phase II study at Wake Forest University is ongoing to test the hypothesis that HIGRT to all known extracranial metastasis following the completion of appropriate systemic therapy can improve outcomes of limited metastatic NSCLC [32]. All patients (with either de novo or recurrent metastases) receive 3 to 6 cycles of systemic therapy at the discretion of the treating medical oncologist and must have stable disease or a partial response. Similar to the University of Chicago study, fractionated therapy can be used to treat stage 3 type intra-thoracic disease. Different from the NCCTG study, hypofractionated image-guided radiotherapy is used which allows for the delivery of metastasis-directed therapy quickly. This study continues to accrue at several centers in North Carolina, USA. Currently, approximately 15 out of a planned 54 patients have been enrolled over the past 18 months.

## 6. Metastasis-Directed HIGRT: A Call to Action

Despite difficulties with accrual in this patient population, there is still a need for randomized studies. The slow accrual of these studies is attributable to shortfalls in study design, and an unfamiliarity among practitioners about the encouraging data is already available for this common problem. Although limited metastatic disease is relatively common, there is considerable heterogeneity with regard to location and number of metastases. Therefore, more flexible radiotherapy dosing schedules are needed. Likewise, flexibility in the systemic therapy is also needed as tailored and targeted regimens gain favor. The currently open Wake Forest trial takes advantage of both of these issues by allowing selection of systemic therapy at the discretion of the treating medical oncologist and selection of the radiotherapy dose based on what the treating radiation oncologist perceives to be achievable. The most commonly used doses on the Wake Forest trial thus far have been 50 Gy in 5 fractions or 50 Gy in 10 fractions prescribed to the PTV margin. 

Perhaps most importantly, study design should reflect thoughtful consideration of the ethical issues surrounding aggressive therapy for metastatic NSCLC. Specifically, studies that prioritize patient and physician equipoise are most likely to meet accrual goals. An ideal trial would register patients during the first two cycles of chemotherapy, but only randomize following restaging showing no evidence of progression. This would allow selection of patients with truly oligometastatic disease where chemotherapy would likely have impacted micrometastatic disease. Patients would then be randomized to (1) HIGRT followed by further systemic therapy or (2) systemic therapy with conventional RT reserved for standard palliative indications. Biologic correlative studies analyzing blood and tissue would be essential. 

Furthermore, emerging data may improve patient selection beyond simple number and location of metastases. Favorable clinical factors such as better performance status [13], limited nodal involvement [65], no prior systemic therapy [28], lack of progression on systemic therapy [28], lack of extracranial metastases [66], metachronous (versus synchronous) brain metastases [67], and 1–3 metastases [28] have been identified. Histologic features such as nonsquamous NSCLC [28, 65] and targetable molecular mutations may also guide patient selection. Serum markers such as low carcinoembryonic antigen level [65] or upregulated Inf-gamma [68] appear to be associated with improved outcomes. An analysis of patients with limited metastases of any histology found that expression of microRNA 200c predicted for true oligometastatic disease with no progression or progression limited in number and destination organs [69]. 

Beyond improved tools to select patients who may derive survival benefits from this therapy, there are other reasons when HIGRT to all known metastatic sites may be beneficial. As noted above, HIGRT is associated with limited toxicity and favorable progression free-survival. For patients unable to tolerate systemic cytotoxic therapy, HIGRT may act as another “line” of therapy. This is an important consideration given the extremely limited activity of second and third line systemic therapies. Alternatively, early evidence suggests HIGRT may sensitize patients to systemic therapy due to the immunomodulatory abscopal effect (as noted above). If confirmed in NSCLC, such an effect could enhance the role of HIGRT for patients with limited or not-so limited metastatic NSCLC. 

Additionally, ideal clinical trials may ultimately need to be developed that test the concept of aggressive local therapy with minimal toxicity in general rather than simply a radiotherapy approach to this disease. This should include combinations of surgery, thermal ablation, HIGRT, radio or chemoembolization, and radiotherapy tailored to each individual patient. Additionally, should patients progress with new sites of disease, metastasis-directed therapies with nonoverlapping toxicity profiles should be considered. This will be particularly useful for patients who have exhausted radiotherapy options due to issues of cumulative dose. In this way patients can be rendered free of macroscopically visible disease and the use of chemotherapy can be appropriately relegated to the goal of the eradication of microscopic disease.

## 7. Conclusion

From the data above it is clear that in appropriately selected patients, aggressive treatment of extracranial metastases and primary tumors can lead to meaningful improvements in overall and progression-free survival. Studies need to be conducted to explore the impact of these therapies. Clearly this will take a coordinated effort. It is unlikely that single cooperative groups will be able to independently accrue and complete these studies. It will take not only an intergroup effort, but also an international effort to complete these studies. The time to conduct these studies is now.

## Figures and Tables

**Table 1:** Selected series for the comprehensive treatment of metastatic NSCLC.

Study	*N*	Metastatic sites	Treatments	1-year PFS	5-year OS
University of Maryland [23]	72	Brain (metachronous)	SRS		13.2%
University of Maryland [24]	42	Brain (synchronous)	SRS, TS, RT, CRT, HIGRT		21%
Hopital Louis Pradel Hospices Civils de Lyon, Lyonnce [25]	51	Brain (synchronous)	BS, TS, RT, CRT		42% (BS + others) versus 5% (BS only)*
University of Rochester [26]	38	Multisite, 1–8 metastases	HIGRT		14%
Rush University Medical Center [27]	23	Multi-site, 1-2 metastases	TS, RT, HIGRT		22%
University of Chicago [28]	25	Multi-site, 1–5 metastases	HIGRT (3–10 fx)	28%	53% (18 mo)
Maastricht University Medical Center [29]	39	Brain, bone, adrenal	TS, SRS, RT, HIGRT		24%*

*2 yr estimates; SRS: stereotactic radiosurgery; BS: brain surgery; TS: thoracic Surgery; RT: radiotherapy; CRT: chemoradiotherapy; HIGRT: hypofractionated image-guided radiotherapy.

**Table 2:** Prospective study characteristics for comprehensive treatment of limited metastatic NSCLC with hypofractionated RT.

Study group	Inclusion	Systemic therapy	Radiotherapy	Outcome
NCCTG [30]	1–3 metastatic sites	Nonstandardized	60 Gy (2 Gy fx) 45 Gy (3 Gy fx)	Closed due to poor accrual
University of Chicago [31]	1–5 metastatic sites	Cisplatin docetaxel	50 Gy (5 Gy fx) 60 Gy (2 Gy fx) if Combined with CT	Closed due to poor accrual
Wake Forest University [32]	Limited metastatic NSCLC	Non-standardized	HIGRT or conventional RT	Open to accrual
